# How does gender influence the recognition of cardiovascular risk and adherence to self-care recommendations?: a study in polish primary care

**DOI:** 10.1186/1471-2296-14-165

**Published:** 2013-11-01

**Authors:** Ireneusz Szymczyk, Ewa Wojtyna, Witold Lukas, Joanna Kępa, Teresa Pawlikowska

**Affiliations:** 1 Department of Family Medicine, Medical University of Silesia, ul. 3 Maja 13/15, 41-800 Zabrze, Poland; 2 Institute of Psychology, University of Silesia in Katowice, ul. Grażyńskiego 53, 40-126 Katowice, Poland; 3 University of Warwick, Coventry, United Kingdom

**Keywords:** Adherence, Cardiovascular risk, Coping, Gender, Health behavior, Stress

## Abstract

**Background:**

Studies have shown a correlation between gender and an ability to change lifestyle to reduce the risk of disease. However, the results of these studies are ambiguous, especially where a healthy lifestyle is concerned. Additionally, health behaviors are strongly modified by culture and the environment. Psychological factors also substantially affect engagement with disease-related lifestyle interventions. This study aimed to examine whether there are differences between men and women in the frequency of health care behavior for the purpose of reducing cardiovascular risk (CVR), as well as cognitive appraisal of this type of risk. We also aimed to identify the psychological predictors of engaging in recommended behavior for reducing the risk of cardiovascular disease after providing information about this risk in men and women.

**Methods:**

A total of 134 consecutive eligible patients in a family practice entered a longitudinal study. At initial consultation, the individual’s CVR and associated health burden was examined, and preventive measures were recommended by the physician. Self-care behavior, cognitive appraisal of risk, and coping styles were then assessed using psychological questionnaires. Six months after the initial data collection, the frequency of subjects’ self-care behavior was examined.

**Results:**

We found an increase in health care behavior after providing information regarding the rate of CVR in both sexes; this increase was greater for women than for men. Women followed self-care guidelines more often than men, particularly for preventive measures and dietary advice. Women were more inclined to recognize their CVR as a challenge. Coping style, cognitive appraisal, age, level of health behaviors at baseline and CVR values accounted for 48% of the variance in adherence to self-care guidelines in women and it was 52% in men. In women, total risk of CVD values were most important, while in men, cognitive appraisal of harm/loss was most important.

**Conclusions:**

Different predictors of acquisition of health behavior are encountered in men and women. Our results suggest that gender-adjusted motivation models influencing the recognition process need to be considered to optimize compliance in patients with CVR.

## Background

Implementing primary prevention, including smoking cessation, healthy diet programs, obesity clinics, and the promotion of exercise, contributes to 25% of the total decline in coronary heart disease-related mortality [1-5]. Studies have also addressed the gender-specific efficacy of particular lifestyle interventions aimed at improving patients’ cardiovascular risk profile [6,7]. They have suggested that cultural and environmental factors could modify this process; in particular for women who, despite their lower cardiovascular risk profile, are in relatively worse physical shape and have a bigger symptom burden than men [8-10].

Among several bio-psychosocial factors influencing an individual’s health behavior pattern, cognitive factors appear to be the most open to discussion, and hence negotiation, in consultation with a physician. These psychological traits may be observed and then modified in the family practice setting to induce better health promotion in patients (e.g., coping style may be modified) [11-14]. However, the effect of social background on the pattern of adopted healthy behavior has been predominantly investigated so far [7-10].

A number of studies have shown a correlation between sex and an ability to change lifestyle to reduce the risk of disease [7,15-22]. However, the results of these studies are ambiguous, especially where healthy lifestyle is concerned. Studies have also highlighted that health behaviors are strongly modified by culture and the environment.

Many studies have shown that women use health care more often than men. This phenomenon is often explained by differences in biological functions related to the sexes. Ailments related to the menstrual cycle, pregnancy, and hormonal changes during menopause may account for the fact that women make use of medical procedures more often than men [7].

Older women, who retire earlier than men, and have been widowed, often use medical institutions as a way of satisfying their needs for social contact. However, previous findings have suggested that women often postpone diagnostics and prevention procedures because of their family duties (e.g., taking care of their children, housekeeping, and simultaneously maintaining an occupation or career) [23,24]. Other studies have suggested that compliance is more often observed in men than in women, and this is attributed to a higher education and material status, as well as their lower burden of a gender role conflict (e.g., cardiovascular prevention with the use of aspirin [17], physical activity [25-27], general cardiovascular risk primary prevention [23], and reduction of obesity [28]). With men being less overloaded by combining occupation and family roles, they may engage more regularly in the above-mentioned types of behavior. Women’s beliefs of being less prone to cardiovascular disease (CVD), which are often triggered by the media, may also account for the above-mentioned differences [7]. In spite of the growing consumption of alcohol and the number of cigarettes smoked by women, in many countries they are still less likely to smoke or drink alcohol than men. Moreover, weak alcohol beverages are dominant in women’s patterns of alcohol use [7,27,29-31]. These differences between sexes are more prominent in difficult situations (e.g., under emotional pressure, men are more willing to drink or smoke cigarettes) [7,29,32].

Self-assessment of one’s health is essential to undertake appropriate preventive action, but findings of studies concerning this are inconclusive [33]. Research describing women’s evaluation of their physical health is contradictory, with reports that regardless of their lower CVD risk, women evaluate their health as poorer than men [7,10,24,34], or in some cases, better than men [35].

Changing lifestyle and maintaining this new state is difficult for individuals. There is a tendency to abstain from continuing healthy behavior if the effort of upholding such activity is higher than one’s own resources [36,37]. This type of situation can focus an individual’s attention on coping with stress and emotions [38,39]. The risk of alcohol or substance abuse, as well as abandoning recommended dietary or other prevention measures, is increased. This behavior is unhealthy, but it generates a positive effect. This behavior temporarily increases the quality of life but it has no effect on medical indicators of wellbeing [38-40]. Therefore, coping is essential to initiate and maintain a healthy lifestyle.

Patients’ reactions to stress appear to mostly depend on cognitive appraisal of the situation. Lazarus and Folkman stated that “Cognitive appraisal is an evaluative process that determines why, and to what extent, a particular transaction or series of transactions between the person and the environment is stressful” [39]. Cognitive appraisal accounts for the first stage of the coping process in which the stressor is recognized and further accommodated for as a challenge, harm/loss, or threat. Therefore, cognitive appraisal determines the coping strategy used [39,41,42]. According to the cognitive behavioral theories regarding the occurrence of emotions and the generation of activities [43-45], cognitive content informs the person’s emotions, as well as his or her behavior. Therefore, appraisal of the stressor in terms of harm/loss is frequently followed by sadness, grief, and passive behavior. In addition, a threat gives rise to anxiety and escape or erratic behavior, while challenge may result in a variety of emotions, including positive emotions such as hope, and these are accompanied by involvement and goal-oriented activities.

Coping style is also affected by numerous personality traits and habits. Inter-sex differences in avoidance styles are adopted. Women participate in social support to address difficult situations, share problems with others, and present a more outgoing attitude towards co-sufferers by revealing their concerns and exchanging experiences more frequently than men [46-51]. Using CVD as an example, once the threat of a cardiovascular event is recognized, cognitive appraisal of the situation and individual coping style substantially affect the pattern of self-care behavior in people. Empirical data are still scarce on the practical implications of psychological determinants for the primary prevention of cardiovascular disorders in men and women.

This study aimed to explore the inter-sex differences in the adopted pattern of self-care behavior for the purpose of reducing cardiovascular risk (CVR), as well as the cognitive appraisal of this risk. Although these differences were presumed at baseline, directional hypotheses were not able to be defined because of the ambiguous results of previous research. Another purpose of the study was to identify the predictors for maintaining healthy behavior targeted at reducing the CVR in women and men.

## Methods

### Participants

A total of 150 patients were successively recruited as they appeared in family practice if they met the following criteria: there were no serious chronic diseases; there was no serious cognitive impairment; patients were aged 40–65 years; and patients consented to participation and follow-up.

**Figure 1:**
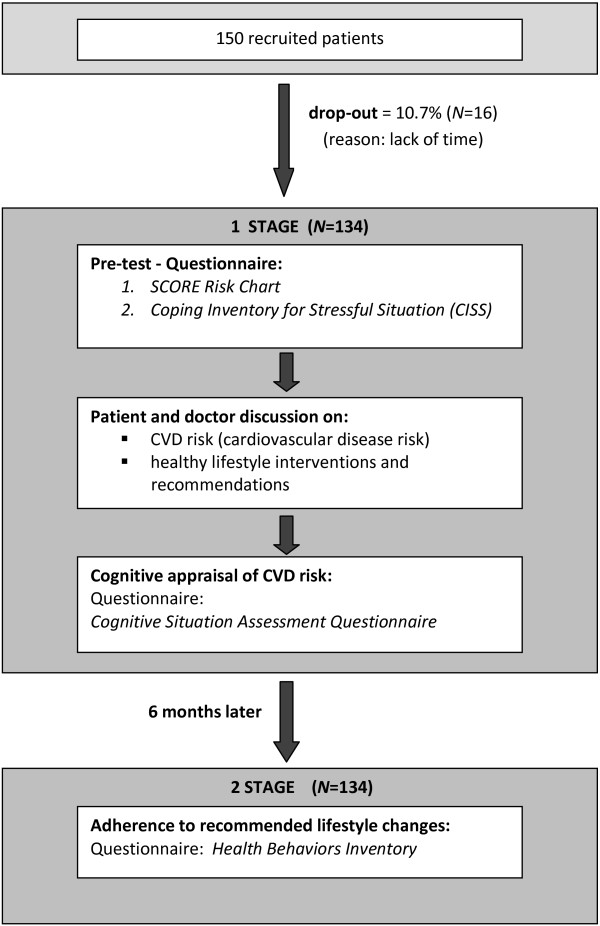
Recruitment process and the study scheme.

Sixteen subjects (10.7%) did not participate in the study because of a lack of time. The process of patients’ recruitment and the scheme of the study are shown in Figure 1. The process of recruiting participants for this study lasted 2 weeks (21 November – 5 December 2011). A total of 134 patients (73 women and 61 men) met all criteria and were included in the study (Table 1). Ethics approval for the study was provided by the Ethics Committee of the University of Silesia.

**Table 1:** Descriptive characteristics of the study participants

**Variable**		**All**	**Males**	**Females**	**Comparison between males and females**
		***N*** **= 134**	***N*** **= 61**	***N*** **= 73**	***p***
Marital status	Single	2.2	1.6	2.7	ns^a^
	Married/Partnership	73.1	73.8	72.6	
[%]	Widow(-er)	10.5	8.2	12.3	
	Separated	14.2	16.4	12.3	
Education	Primary	2.2	1.6	2.7	ns^a^
[%]	Vocational	23.1	19.6	26.0	
	Secondary	50.8	47.5	53.4	
	University	23.9	31.2	17.8	
Age [Mean (SD)]		56.67 (9.29)	55.02 (9.02)	54.42 (9.53)	ns^b^
Risk of CVD (SCORE)	Low	54.2	28.9	72.6	
	Moderate	22.4	35.6	12.9	.001^a^
[%]	High	23.4	35.6	14.5	

*Note*: a – chi-squared test; b – Student’s t-test; ns – statistically non-significant.

### Data collection

#### Study design and procedure

This prospective study consisted of two parts (Figure 1). The first stage of the study focused on the patient’s coping style, as well as the level of health activity assessment. The Polish version of the Coping Inventory for Stressful Situations (CISS) [41] and Health Behavior Inventory (HBI) [52] were used. Having obtained the participants’ consent, the following measurements were performed: systolic blood pressure, total cholesterol level, and high-density lipoprotein. The participants were then asked to complete Polish versions of the CISS and HBI. Having obtained laboratory data, the risk of CVD for each patient was calculated using the Systematic Coronary Risk Evaluation (SCORE) Chart [53]. All questionnaire data were collected by a resident or a psychologist.

Doctors talked to patients about their predicted CVD risk. They discussed some appropriate changes in the patient’s lifestyle. This discussion lasted approximately 30 minutes and was carried out by the same physician (I.S.). Finally, immediately after this consultation, patients were asked to fill in a questionnaire on cognitive appraisal of CVD risk assessment [42].

The second phase of the study took place 6 months after the initial data collection. The researchers responsible for data collection (J.K. and E.W.) invited patients by a telephone call to visit the family practice to complete questionnaires. Forty-one subjects could not participate at the family practice, and therefore they were asked to answer all of the questions by phone. Data were collected from all 134 participants recruited in the first phase of the study. Patients were asked to fill in a questionnaire on recommended health behavior (HBI) (Figure 1).

#### Assessment tools

##### CVD risk assessment

The Polish version of the SCORE Risk Chart was used [53]. This chart is recommended by the European Society of Cardiology for calculating the absolute 10-year probability of developing a fatal cardiovascular event. The SCORE Risk Chart takes the following factors into consideration: sex, age, smoking status, systolic blood pressure, and the total cholesterol/high-density lipoprotein ratio. The SCORE Risk Chart allowed participants to be categorized into one of the following risk groups: low (<3%), medium (3–4%), or high (≥5%).

##### Health behavior

The reported pattern of self-care behavior adopted was determined using the Health Behaviors Inventory (HBI) [52]. This questionnaire comprises 24 items and covers four different categories of behavior: dietary self-management, preventive measures, healthy practices, and positive mental attitude.

##### Cognitive appraisal of stressors and coping

The Cognitive Situation Assessment Questionnaire was used to examine cognitive appraisal parameters [42]. Study participants were given a version addressing patient recognition of their chances of developing fatal CVD. This questionnaire comprises 35 items and can be used to explore cognitive appraisal parameters. Items are grouped into different categories of challenge, harm/loss, and threat.

The Polish version of the Coping Inventory for Stressful Situation (CISS) [41,54] was used to determine each participant’s coping style within the following three different categories: (1) The task-centered style is characterized by concentrating one’s attention and efforts on attempts to find a solution for a certain problem. In the case of being at risk of a culprit lesion, this style may induce diet changes, introducing physical activity, or active searching for information regarding possibilities of improving one’s health (2). The emotion-centered style is characterized by concentrating on one’s emotions, such as anger, anxiety, or guilt. In stressful situations, this category might induce wishful thinking and fantasizing aimed at reducing any tension experienced. However, this category may also be linked with refraining from activities that are important to one’s health, or even engaging in non-healthy behavior (e.g., smoking or excessive consumption of alcohol) (3). The avoidance-centered style is characterized by a tendency to avoid thinking, experiencing, or living through a threatening situation. As a result, the necessity of resolving a health problem is postponed in time. This process might be as follows: engaging in substitute actions (i.e., excessive eating, distracting one’s attention, engaging in thinking about pleasant matters, watching television more than usual, reading books on neutral topics, and excessive sleepiness); and seeking social contacts. The CISS comprises 48 items assessed with the Likert scale.

#### Data analysis

Statistical analysis was carried out using SPSS version 21.0. Descriptive data are presented as means and standard deviations for continuous data and percentages for categorical data. To examine differences in baseline characteristics (descriptive data, cognitive appraisal of CVD risk, and style of coping with stress) between men and women, the Student’s *t-*test and the chi-squared test were used.

To determine whether gender and possession of information about the possible risk of CVD have an effect on engaging in health behavior, a multivariate repeated-measures ANOVA was conducted. The dependent variable was the frequency of health behavior 6 months after having received information on the rate of risk for CVD and the independent variables were gender and state of knowledge about CVR (before and after information concerning CVD risk).

To explore relationships between psychological factors, such as cognitive appraisal of the CVD risk and coping style, and engaging in health behavior, Pearson’s r correlation coefficient was calculated.

To identify predictors of engaging in healthy behavior in women and men, multiple regression analysis was conducted. The dependent variable was the change in frequency of healthy behavior aimed at reducing the risk of CVD, which participants engaged in 6 months after having received information about the CVR. This change was defined as a difference between the frequency of healthy behavior post- and pretest. The following variables were entered as independent variables: age, health behavior at baseline, SCORE at baseline, cognitive appraisal of risk, and coping style. Regression analyses were conducted separately for men and women.

## Results

CVD risk in men was significantly higher (p < 0.001) than that in women (Table 1). A total of 71.1% of men had at least a moderate CVD risk, while in women, over two-thirds were in the low-risk group.

Women and men treated information about their risk of fatal CVD mostly as a challenge. Some of them considered this risk a harm/loss and only a few took it as a threat. Comparison by *t*-test between men and women showed statistically significant differences only in the challenge category (p < 0.01). Women were more inclined than men to recognize their CVR as a challenge (Table 2). Remaining categories of the cognitive appraisal of CVD risk presented with equal frequency in men and women. No difference was observed between men and women with regard to coping styles.

**Table 2:** Cognitive appraisal of CVR and style of coping regarding gender

**Stress**	***Gender [mean (SD)]***		***t***
	**Females**	**Males**	
Cognitive appraisal of CVD risk			
Threat	17.98 (5.81)	18.77 (6.20)	0.65
Harm/loss	9.96 (2.52)	9.61 (1.84)	0.83
Challenge	36.04 (6.14)	32.83 (5.75)	3.98**
Style of coping			
Task-oriented	57.65 (8.05)	59.31 (6.13)	1.18
Emotion-oriented	42.83 (9.02)	42.22 (8.95)	0.28
Avoidant	45.60 (6.73)	44.38 (9.23)	0.65

*Note*: **p < .01 Student’s *t-*test.

The level of adherence to self-care recommendations before and after 6 months since the discussion on CVD risk, taking gender into account, is shown in Table 3. In multivariate repeated-measures ANOVA, several effects were found to be significant: there was a main effect of gender (eta^2^ = 0.12; p < 0.001), a main effect of information about CVD risk (eta^2^ = 0.13; p < 0.01), and an interaction effect of gender and information about CVD risk (eta^2^ = 0.19; p < 0.001). Women engaged in health care behavior more than men. Information on CVR caused women and men to increase their engagement in health care behavior. This observed interaction effect suggests that when women receive information about CVR, they introduce bigger changes in health care behavior than men.

**Table 3:** The risk of CVD related to changes in adherence to health behavior by sex

**Health behaviors**	**Gender**	**Information about CVD risk**		**Sources of variance** ***[F]***		
		***[Mean (SD)]***				
		**Before information**	**After information**	**Gender**	**Info**	Gender*Info
All behaviors	M	74.21 (15.30)	77.73 (14.02)	11.99***	8.32**	12.24***
	F	76.37 (14.46)	89.36 (12.12)			
Dietary self-management	M	16.87 (4.67)	18.09 (4.33)	10.23**	7.13*	10.76***
	F	16.96 (3.05)	21.28 (3.93)			
Preventive measures	M	17.84 (3.85)	19.52 (4.01)	10.05**	6.08*	7.82**
	F	18.98 (3.76)	23.83 (4.98)			
Healthy practices	M	16.92 (4.12)	18.98 (4.65)	4.14*	5.47*	2.26
	F	18.93 (4.39)	20.82 (4.50)			
Positive mental attitude	M	20.57 (4.64)	20.82 (4.09)	5.21*	1.98	3.36*
	F	21.49 (3.53)	23.09 (3.87)			

*Note*: M – men (n = 61); F – females (n = 73); Info – information about CVD risk; *p < .05; **p < .01; ***p < .001; multivariate repeated-measures ANOVA.

In women, there was an overall adherence to self-care regimens, particularly with respect to preventive measures, which were associated with an avoidance-centered coping style (Table 4). In men, there was a positive correlation (p < 0.01) between recognition of CVR as a challenge and adherence to preventive measures. Regardless of gender, elderly people in this study were more inclined to adhere to self-care recommendations.

**Table 4:** Correlation between adopted health behavior and selected psychological factors with regard to gender

**Factors**	**Health behaviors (total)**		**Dietary self-management**		**Preventive measures**		**Positive mental attitude**		**Healthy practices**	
	**F**	**M**	**F**	**M**	**F**	**M**	**F**	**M**	**F**	**M**
Task-oriented coping style	.43*	-.23	.39*	-.13	.44**	-.27	.29	-.19	-.06	-.18
Emotion-oriented coping style	-.01	.03	-.02	.04	.11	.24	-.16	.00	-.11	-.19
Avoidant coping style	.38*	.19	.05	.10	.48**	.40*	.36	.13	.06	-.02
Cognitive appraisal-threat	-.34*	-.02	-.38*	-.22	-.18	.08	-.28	.10	-.29	.00
Cognitive appraisal-harm/loss	-.32*	.02	-.37*	-.03	-.16	.06	-.27	.01	-.25	.02
Cognitive appraisal-challenge	.22	.46*	.17	.11	.20	.56**	.23	.51**	.03	.29
Cardiovascular risk	.24	.27	.01	.22	.21	.20	.28	.22	.17	.23
Age	.32*	.36*	-.01	.22	.22	.35*	.32*	.31*	.41***	.27
Education	.01	.07	.12	.08	-.01	-.03	-.10	.09	-.05	.14

*Note*: *N* = 134; F – females (*N* = 73); M – males (*N* = 61); *p < .05; **p < .01; ***p < .001; Pearson correlations coefficients.

A multiple regression model in men accounted for 52% of variance in the acquisition of healthy behavior (Table 5). Only health behavior at baseline and cognitive appraisal of a situation as a harm/loss event was significantly correlated with men’s adherence to self-care guidelines. In women, 48% of the variance in adherence to self-care guidelines was accounted for by the dimensions of cognitive appraisal, coping style, age, health behavior before information about CVD risk and CVR (Table 5). Considering the predictive strength of each factor, better adherence to healthy behavior was significantly associated with total CVR values alone and higher level of health behavior at baseline.

**Table 5:** Factors affecting the change of adherence to self-care guidelines in men and women with established CVRs

**Factors affecting change of health behavior**	***Men (N = 61)***			***Women (N = 73)***		
	***adj.R***^***2***^ ***= 0.52***			***adj.R***^***2***^ ***= 0.48***		
	***β***	***SE***	***p value***	***β***	***SE***	***p value***
Age	0.03	0.24	0.876	-0.12	0.23	0.499
Health behaviors at baseline	0.79	0.15	<0.001	0.55	0.12	<0.001
Cognitive appraisal						
Threat	-0.01	0.34	0.969	-0.14	0.26	0.416
Harm/loss	0.59	0.16	<0.001	-0.37	0.97	0.072
Challenge	-0.04	0.78	0.794	0.15	0.79	0.324
Coping style						
Task-centered	-0.07	0.30	0.571	-0.07	0.21	0.627
Emotion-centered	0.01	0.24	0.997	-0.28	0.21	0.070
Avoidance-centered	-0.10	0.20	0.444	0.14	0.26	0.311
Total CVD risk estimate (SCORE)	-0.12	2.69	0.417	0.57	0.41	0.005

*Note:* Multiple linear regression.

## Discussion

This study showed gender differences in the pattern of healthy behavior adopted by patients targeted for reduction of CVR in Polish general practice. Women adhered more strictly to the doctor’s recommendations than men. Several cultural factors may support this finding. In the Polish collective memory, there is a strong stereotype of the “Polish Mother” (“Matka Polka”), a woman in charge of the family and household prosperity [55,56]. Although contemporary Polish women successfully compete with men in the workplace, striving to reconcile household activities with job requirements, they are still expected to deny their own ambitions and abandon prestigious jobs to support their husband’s and children’s personal development and careers [24,55,56]. This somewhat conservative model of distinct gender roles is clearly reflected in almost every aspect of contemporary life, and implemented from early childhood and in primary education [55-58].

This conservative attitude (relative to some western EU countries) is deeply rooted among parents and teachers who tend to attribute different personality traits to boys and girls, thereby imposing distinct needs and values on them. Therefore, daughters are taught to be submissive, ready to make sacrifices as good mothers and wives. At the same time, boys are brought up in a competitive spirit, coping with adversity, focusing on career advancement and success in public life. In this process, despite women’s relatively higher education, better job qualifications, and rising participation in the workplace, as much as 80% of women focus on their household and family commitments as their natural and single social activity [55,56].

Such a submissive attitude could account for women’s greater adherence to self-care recommendations. A further interpretation is that the “Polish Mother” strives to stay in good health to maintain her protective function towards her children, and ensures her husband is not deprived of support. This interpretation supports our findings concerning women’s more frequent appraisal of CVR as a challenge. The same applies to CVR itself, which was the main predictor of adherence to self-care behavior in women in our study. The “Polish Mother” seeks self-care, possibly because her family is in danger. Our study is consistent with other Polish reports concerning women’s better self-rated compliance with a doctor’s instructions [27,59].

Our study showed that an avoidant style is associated with preventive measures particularly with women who also undertake healthy behavior more frequently. The avoidant style itself is characterized by seeking and building closer social relations, which in turn serve as a provider of mutual support and relieve the personal stress associated with health hazards [7,47,60]. As a result, when women have their emotional state moderated, they are then much more likely [27] to adopt a recommended pattern of health behavior.

Our study showed that cognitive appraisal of harm or loss was a predictor of adherence to self-care regimens in men. Interpreting this from an analogous perspective of the influence of cultural background, men in Poland take establishing a higher position in society for granted. In the common thinking of Polish people, unemployment status or redundancy does not devalue women, who still have a highly esteemed position in their families, but it severely degrades men. Because of the relative stability of men’s social rank, they are rarely forced to strive for better control of their opportunities for social lives. Therefore, the potentially unexpected loss of these intrinsic resources prompts them to act strongly to counter that potential risk. In contrast to women, men in Poland attribute more importance to the quality of current life, rather than health in old age [7]. Therefore, the sense of deprivation (cognitive appraisal of harm/loss) and recognition of a potentially poorer life and work prospects appear to motivate men to conduct healthy behavior to maintain their current life.

For elderly people, their better adherence to self-care recommendations may be explained by the common association of health care centers with social support providers for them [7]. Faced with deterioration of their support network, elderly people visit their family physician to provide more opportunity for interaction. However, this model appears to imply that such patients have at least minimal compliance with their doctor’s recommendations.

### Limitations

This Polish study was carried out on patients of a single family practice, managed by an organized team comprising family physicians, a psychiatrist, and a clinical psychologist with experience in guidance on preventive programs. Further study with a larger sample of patients in diverse environments is required. The self-rated questionnaires used in this study may be a source of respondent bias. However, these questionnaires were chosen because they are reliable and validated for evaluating adherence to self-care recommendations. Gender can be thought of as an aggregated variable [61]; namely, as encompassing many other variables and values that were not separately accounted for in this study. Controlling for psychological sex, a more specific mental property, and other elements could allow expansion of our study in a broader population.

### Practice implications

Our study has several practice implications concerning the motivation process for adopting healthy behavior in a Polish general practice. Other studies [62,63] have shown that better results are achieved when doctors adjust the type and quantity of information communicated to patients according to an individual’s coping style. Our study implies that a parallel exists in gender-specific expectations towards the intervention studied with regard to the specific cultural setting. Our study suggests that for women, CVR needs to be emphasized together with possible preventive measures based on inherent resources (the recognition of risk as a challenge). A particular approach is via family responsibility, as well as the support of a peer network. Our study implies that men consider important the realization of an impaired quality of current or future life with adverse implications for social participation, career prospects, or sexual health.

## Conclusions

Our study shows that there are different predictors for acquiring healthy behavior in men and women. In women, recognition of total risk is of the highest consequence, while in men, it is cognitive appraisal of harm/loss. This finding implies that sex-adjusted motivation models need to be considered to optimize patient engagement. Further investigation is required concerning sex differences in CVR appraisal and its effect on adherence to preventive measures in different cultural environments.

## Abbreviations

CISS: Coping inventory for stressful situation; CVD: Cardiovascular disease; CVR: Cardiovascular risk; HBI: Health behavior inventory; SCORE: Systematic coronary risk evaluation.

## Competing interests

The authors declare that they have no competing interests.

## Authors’ contributions

IS participated in the design of the study, conducted interviews and examinations, and drafted the manuscript. EW participated in the design of the study, conducted analysis, and contributed to the interpretation of data and drafting of the manuscript. EW, WL, and JK conceived the study. WL, JK, and TP participated in design and discussions, and contributed to drafting of the manuscript. All authors read and approved the final manuscript.

## Pre-publication history

The pre-publication history for this paper can be accessed here:

http://www.biomedcentral.com/1471-2296/14/165/prepub

## References

[B1] Hunink MG Goldman L Tosteson AN Mittleman MA Goldman PA Williams LW Tsevat J Weinstein MC The recent decline in mortality from coronary heart disease, 1980–1990. The effect of secular trends in risk factors and treatment JAMA 1997 227 535 542 9032159

[B2] Dwyer JH Exposure to environmental tobacco smoke and coronary risk Circulation 1997 96 1403 1407 10.1161/01.CIR.96.5.1403 9315516

[B3] Law MR Wald NJ Wu T Hackshaw A Bailey A Systematic underestimation of association between serum cholesterol concentration and ischaemic heart disease in observational studies: data from the BUPA study BMJ 1994 308 363 366 10.1136/bmj.308.6925.363 8124143 PMC2539480

[B4] Polskie Towarzystwo Kardiologiczne [Polish Heart Association] Standardy postępowania w chorobach układu krążenia [Guidelines for Circulatory Disorders] Kardiol Pol 1997 46 Suppl 1

[B5] Goldbourt U Physical activity, long-term CHD mortality and longevity: a review of studies over the last 30 years World Rev Nutr Diet 1997 82 229 239 9270325 10.1159/000059644

[B6] Rodin J Salovey P Heszen-Niejodek I, Sęk H Psychologia zdrowia [Psychology of Health] Psychologia zdrowia [Psychology of Health] 1997 Warsaw: PWN 62 109

[B7] Ostrowska A Styl życia a zdrowie [Lifestyle and Health] 1999 IFiS PA: Warsaw

[B8] Brunner RL Understanding gender factors affecting self-rated health Gend Med 2006 3 292 294 10.1016/S1550-8579(06)80217-2 17582370

[B9] Tobiasz-Adamczyk B Wybrane elementy jakości życia kobiet starszych wiekiem [Selected elements of the quality of life of older women] Promocja Zdrowia, Nauki Społeczne i Medycyna [Health Promotion, Social Science and Medicine] 1999 6 68 83

[B10] Lim WY Ma S Heng D Bhalla V Chew SK Gender, ethnicity, health behaviour and self-rated health in Singapore BMC Public Health 2007 7 184 10.1186/1471-2458-7-184 17655774 PMC1976324

[B11] Leung MY Alexander M Chun CJP Coping and adjustment in Chinese patients with chronic obstructive pulmonary disease Int J Nurs Stud 2002 39 383 395 10.1016/S0020-7489(01)00036-0 11909615

[B12] Malan L Schutte AE Malan NT Wissing MP Vorster HH Steyn HS van Rooyen JM Huisman HW Coping mechanisms, perception of health and cardiovascular dysfunction in Africans Int J Psychophysiol 2006 61 158 166 10.1016/j.ijpsycho.2005.07.015 16257466

[B13] Hamer M Malan L Psychophysiological risk markers of cardiovascular disease Neurosci Biobehav Rev 2010 35 76 83 10.1016/j.neubiorev.2009.11.004 19909773

[B14] Gleiberman L Repressive/defensive coping, blood pressure, and cardiovascular rehabilitation Curr Hypertens Rep 2007 9 7 12 10.1007/s11906-007-0003-9 17362665

[B15] Wood FG Ethnic differences in exercise among adults with diabetes West J Nurs Res 2002 24 502 515 10.1177/019394502400446388 12148832

[B16] Ward KD Vander Weg MW Kovach KW Klesges RC DeBon MW Haddock CK Talcott GW Lando HA Ethnic and gender differences in smoking and smoking cessation in a population of young adult Air Force recruits Am J Health Promot 2002 16 259 266 10.4278/0890-1171-16.5.259 12053437

[B17] Opotowsky AR McWilliams JM Cannon CP Gender differences in aspirin use among adults with coronary heart disease in the United States J Gen Intern Med 2007 22 55 61 10.1007/s11606-007-0116-5 17351840 PMC1824779

[B18] Trudeau L Spoth R Randall G Azevedo K Longitudinal effects of a universal family-focused intervention on growth patterns of adolescent internalizing symptoms and polysubstance use: gender comparisons J Youth Adolesc 2007 36 725 740 10.1007/s10964-007-9179-1

[B19] Azevedo MR Araújo CL Reichert FF Siqueira FV da Silva MC Hallal PC Gender differences in leisure-time physical activity Int J Public Health 2007 52 8 15 10.1007/s00038-006-5062-1 17966815 PMC2778720

[B20] Gibbons SW Barnett SD Hickling EJ Herbig‒Wall PL Watts DD Stress, coping, and mental health‒seeking behaviors: gender differences in OEF/OIF health care providers J Trauma Stress 2012 25 115 119 10.1002/jts.21661 22354515

[B21] Polen MR Green CA Perrin NA Anderson BM Weisner CM Drinking patterns, gender and health: attitudes and health practices Addict Res Theory 2010 18 122 142 10.3109/16066350903398486 23946720 PMC3740444

[B22] Schneider U Pfarr C Schneider BS Ulrich V I feel good! Gender differences and reporting heterogeneity in self-assessed health Eur J Health Econ 2012 13 251 265 10.1007/s10198-011-0301-7 21305334

[B23] King KM Arthur HM Coronary heart disease prevention. Views on women’s gender-based perception and meanings J Cardiovasc Nurs 2003 4 274 281 14518603 10.1097/00005082-200309000-00006

[B24] Fleury J Keller C Murdaugh C Social and contextual etiology of coronary heart disease in women J Womens Health Gend Based Med 2000 9 967 978 10.1089/15246090050199991 11103096

[B25] Blanchard CM Rodgers WM Courneya KS Daub B Black B Self-efficacy and mood in cardiac rehabilitation: should gender be considered? Behav Med 2002 27 149 160 10.1080/08964280209596040 12165969

[B26] Navuluri RB Gender differences in the factors related to physical activity among adults with diabetes Nurs Health Sci 2000 2 191 199 10.1046/j.1442-2018.2000.00056.x

[B27] Wojtyna E Poznawcze, afektywne i społeczne uwarunkowania stosowania się do zaleceń lekarskich przez chorych na cukrzycę typu 2 [Cognitive, affective and social predictors of compliance among patients with diabetes mellitus type 2] PhD thesis 2012 University of Silesia in Katowice, Department of Education and Psychology

[B28] Bautista-Castaño I Molina-Cabrillana J Montoya-Alonso JA Serra-Majem L Variables predictive of adherence to diet and physical activity recommendations in the treatment of obesity and overweight, in a group of Spanish subjects Int J Obes 2004 28 697 705 10.1038/sj.ijo.0802602 14993911

[B29] McCreary DR Newcomb MD Sadava SW The male role, alcohol use, and alcohol problems: a structural modeling examination in adult women and men J Couns Psychol 1990 46 109 124

[B30] Li Y Jiang Y Zhang M Yin P Wu F Zhao W Drinking behaviour among men and women in China: the 2007 China Chronic Disease and Risk Factor Surveillance Addiction 2011 106 1946 1956 10.1111/j.1360-0443.2011.03514.x 21771141

[B31] Roberts SCM Whether men or women are responsible for the size of gender gap in alcohol consumption depends on alcohol measure: a study across the United States Contemp Drug Probl 2012 39 195 212 23248388 10.1177/009145091203900202 PMC3522467

[B32] Babwah F Baksh S Blake L Cupid-Thuesday J Hosein I Sookhai A Poon-King C Hutchinson G The role of gender in compliance and attendance at an outpatient clinic for type 2 diabetes mellitus in Trinidad Rev Panam Salud Publica 2006 19 79 84 16551381 10.1590/s1020-49892006000200002

[B33] Lusk SL Ronis DL Kerr MJ Atwood JR Test of the health promotion model as a causal model for workers’ use of hearing protection Nurs Res 1994 43 151 157 8183656

[B34] Ahmad K Jafar TH Chaturvedi N Self-rated health in Pakistan: results of a national health survey BMC Public Health 2005 19 51 15943882 10.1186/1471-2458-5-51 PMC1164420

[B35] Leinsalu M Social variation in self-rated health in Estonia: a cross-sectional study Soc Sci Med 2002 55 847 861 10.1016/S0277-9536(01)00221-0 12190275

[B36] Hobfoll S Conservation of resources. A new attempt at conceptualizing stress Am Psychol 1989 44 513 524 2648906 10.1037//0003-066x.44.3.513

[B37] Hobfoll S Stress, culture and community: The psychology and philosophy of stress 1998 New York: Plenum Press

[B38] Endler NS Parker JDA Summerfeldt LJ Coping with health problems: developing a reliable and valid multidimensional measure Psychol Assess 1998 10 195 205

[B39] Lazarus R Folkman S Stress, appraisal and coping 1984 New York: Springer-Verlag

[B40] Folkman S Lazarus R Coping as mediator of emotion J Pers Soc Psychol 1984 54 466 475 3361419

[B41] Endler NS Parker JDA Assessment of multidimensional coping: task, emotion and avoidance strategies Psychol Assess 1994 6 50 60

[B42] Włodarczyk D Rola i miejsce oceny poznawczej w radzeniu sobie ze stresem [The association between cognitive assessment and coping] Nowiny Psychologiczne [Psychological News] 1999 4 57 72

[B43] Ellis A Dryden W The practice of rational emotive behavior therapy 2007 2 New York: Springer Publishing Company

[B44] Maultsby MC Rational behavior therapy 1990 Rational Self-Help Books/I’ACT: Appleton

[B45] Clark DME Fairburn CG Science and practice of cognitive behaviour therapy 1997 Oxford: Oxford University Press

[B46] Lopez CR Antoni MH Fekete EM Penedo FJ Ethnic identity and perceived stress in HIV+ minority women: the role of coping self-efficacy and social support Int J Behav Med 2012 19 23 28 10.1007/s12529-010-9121-x 20890774

[B47] Day AL Livingstone HA Gender differences in perceptions of stressors and utilization of social support among university students Can J Behav Sci 2003 35 73 83

[B48] Ptacek JT Smith RE Dodge KL Gender differences in coping with stress: when stressors and appraisal do not differ Pers Soc Psychol Bull 1994 20 421 430 10.1177/0146167294204009

[B49] Krajewski HT Goffin RD Predicting occupational coping responses: the interactive effect of gender and work stressors context J Occup Health Psychol 2005 10 44 53 15656720 10.1037/1076-8998.10.1.44

[B50] Tamres LK Janicki D Helgeson VS Sex differences in coping behavior: a meta-analytic review and an examination of relative coping Pers Soc Psychol Rev 2002 6 2 30 10.1207/S15327957PSPR0601_1

[B51] Greenglass ER Burke RJ Work and family precursors of burnout in teachers: sex differences Sex Roles 1988 18 215 229 10.1007/BF00287791

[B52] Juczyński Z Narzędzia pomiaru w promocji i psychologii zdrowia [Assessment tools in health promotion and psychology] 2001 Pracownia Testów Psychologicznych: Warsaw

[B53] Conroy RM Pyorala K Fitzgerald AP Sans S Menotti A De Backer G De Bacquer D Ducimetiere P Jousilahti P Keil U Njolstad I Oganov RG Thomsen T Tunstall-Pedoe H Tverdal A Wedel H Whincup P Wilhelmsen L Graham IM Estimation of ten-year risk of fatal cardiovascular disease in Europe: the SCORE project Eur Heart J 2003 24 987 1003 10.1016/S0195-668X(03)00114-3 12788299

[B54] Szczepaniak P Strelau J Wrześniewski K Diagnoza stylów radzenia sobie ze stresem za pomocą polskiej wersji kwestionariusza CISS Endlera i Parkera [Diagnosis of coping styles based on Polish version of CISS questionnaire by Endler & Parker] Przegląd Psychologiczny [Psychological Review] 1996 39 187 210

[B55] Kobiet RCP Kobiety w Polsce w latach 90-tych [Polish Women in 90s. The Report of Centre for Women’s Rights] 2000 Warsaw: Fundacja Centrum Praw Kobiet

[B56] Oleksy EH Selected proceedings of the Women's Studies Conference, Łódź, Poland, May 17–21 Womens Stud Int Forum 1995 18 3 8

[B57] Firkowska-Mankiewicz A Titkow A, Domański H Czy tak samo wychowujemy dzieci [Are our children being brought up in the same way]? Co to znaczy być kobietą w Polsce [What does it mean to be a woman in Poland] 1995 Warsaw: PAN

[B58] Dabic M Women and Academic Careers in Poland Equality and Partnership towards Higher Education, Employment/Entrepreneurship and Environmental Management in Central and Eastern European Countries. Future Strategic Goals and Objectives. Conference Publication, 1–3 September 1994 1994 Wolfheze, Holland

[B59] Łopuszańska M Szklarska A Jankowska EA Zachowania zdrowotne dorosłych mężczyzn i kobiet w Polsce w latach 1984 i 1999 [Health Behaviours of adult men and women in Poland in 1984 and 1999 year] Zdrowie Publiczne [Public Health] 2004 114 23 28

[B60] Viswesvaran C Sanchez JL Fisher J The role of social support in the process of work stress: a meta-analysis J Vocat Behav 1999 54 314 334 10.1006/jvbe.1998.1661

[B61] Spendel Z Popiołek K, Bańka A O pewnych niebezpieczeństwach nadużywania etykiet zastępczych. Niespecyficzne Zmienne Zagregowane (NZZ) w badaniach psychologicznych [About some dangers concerning overusing of substitution labels. Non-specific Aggregated Variables] Kryzysy, katastrofy, kataklizmy w kontekście narastania zagrożeń [Crises, catastrophes and disasters with regard to overwhelming threats] 2007 Poznań: Stowarzyszenie Psychologia i Architektura

[B62] Ludwick-Rosenthal R Neufeld WJ Preparation for undergoing an invasive medical procedure: interacting effects of information and coping style J Consult Clin Psychol 1993 61 156 164 8450101 10.1037/0022-006X.61.1.156

[B63] Miller SM Mangan CE The interacting effects of information and coping style in adapting to gynecologic stress: should the doctor tell all? J Pers Soc Psychol 1983 45 223 236 6886967 10.1037//0022-3514.45.1.223

